# Hydrogen-Bonded Structure of Water in the Loop of Anchored Polyrotaxane Chain Controlled by Anchoring Density

**DOI:** 10.3389/fchem.2021.743255

**Published:** 2021-10-18

**Authors:** Keishi Akada, Kosuke Yamazoe, Jun Miyawaki, Rina Maeda, Kohzo Ito, Yoshihisa Harada

**Affiliations:** ^1^ Institute for Solid State Physics (ISSP), The University of Tokyo, Chiba, Japan; ^2^ Department of Advanced Materials Science, Graduate School of Frontier Sciences, The University of Tokyo, Chiba, Japan; ^3^ Synchrotron Radiation Research Organization, The University of Tokyo, Chiba, Japan

**Keywords:** polyrotaxane, PEG, polymer loop, biocompatibility, soft X-ray emission

## Abstract

Hydrogen-bonded network of water surrounding polymers is expected to be one of the most relevant factors affecting biocompatibility, while the specific hydrogen-bonded structure of water responsible for biocompatibility is still under debate. Here we study the hydrogen-bonded structure of water in a loop-shaped poly(ethylene glycol) chain in a polyrotaxane using synchrotron soft X-ray emission spectroscopy. By changing the density of anchoring molecules, hydrogen-bonded structure of water confined in the poly(ethylene glycol) loop was identified. The XES profile of the confined water indicates the absence of the low energy lone-pair peak, probably because the limited space of the polymer loop entropically inhibits the formation of tetrahedrally coordinated water. The volume of the confined water can be changed by the anchoring density, which implies the ability to control the biocompatibility of loop-shaped polymers.

## Introduction

Water is well known to play an important role in most biological processes by mediating specific functions, such as biocompatibility of biomaterials (Bag and Valenzuela, 2017; Laage et al., 2017). When a biomolecule approaches the surface of a biomaterial, a few layers of water molecules surrounding both interacting media, called hydration shells, directly interact with each other and regulate the adsorption or desorption property. Many researchers have proposed various models for the hydrogen-bonded structure of the hydration shell of biomaterials (Kitano et al., 2000; Asay and Kim, 2005; Morita et al., 2007). However, much debate still exists on the structure of water that provides biocompatibility and how to design biomaterials to improve this function (Jena and Hore, 2010; Tanaka and Mochizuki, 2010; Bag and Valenzuela, 2017; Laage et al., 2017). Indeed, biocompatibility can also be improved by the morphology of the polymer in addition to its constituents. For example, an excellent anti-fouling property [hereafter referred to as biocompatibility because this is often linked to nonbiofouling (Schlenoff, 2014)] has been reported for looped polymers (Yamada et al., 2012; Kang et al., 2016; Benetti et al., 2017). Articular joints of mammalians are coated with a lubricating protein, which adopts the looped conformation, has also exhibited outstanding biocompatibility (Greene et al., 2015). The looped shape is more rigid and provides stronger resistance against compression than a linear one. The higher resistance of the looped chains towards external compression translated into an excellent biocompatibility, a protein repellent behavior (Yamada et al., 2012; Benetti et al., 2017). The looped shape also accommodates water molecules inside loops having a size of a few nanometers (Ostaci et al., 2011). Water encapsulated within such a small space, e.g., in mesoporous silica, has changed freezing and melting points much lower than those of bulk water (Kittaka et al., 2006). This property is called cold crystallization, which is commonly observed in biocompatible materials like poly (methoxyethyl acrylate) (PMEA), one of the most practical biocompatible polymer materials (Tanaka and Mochizuki, 2010).

**SCHEME 1:**
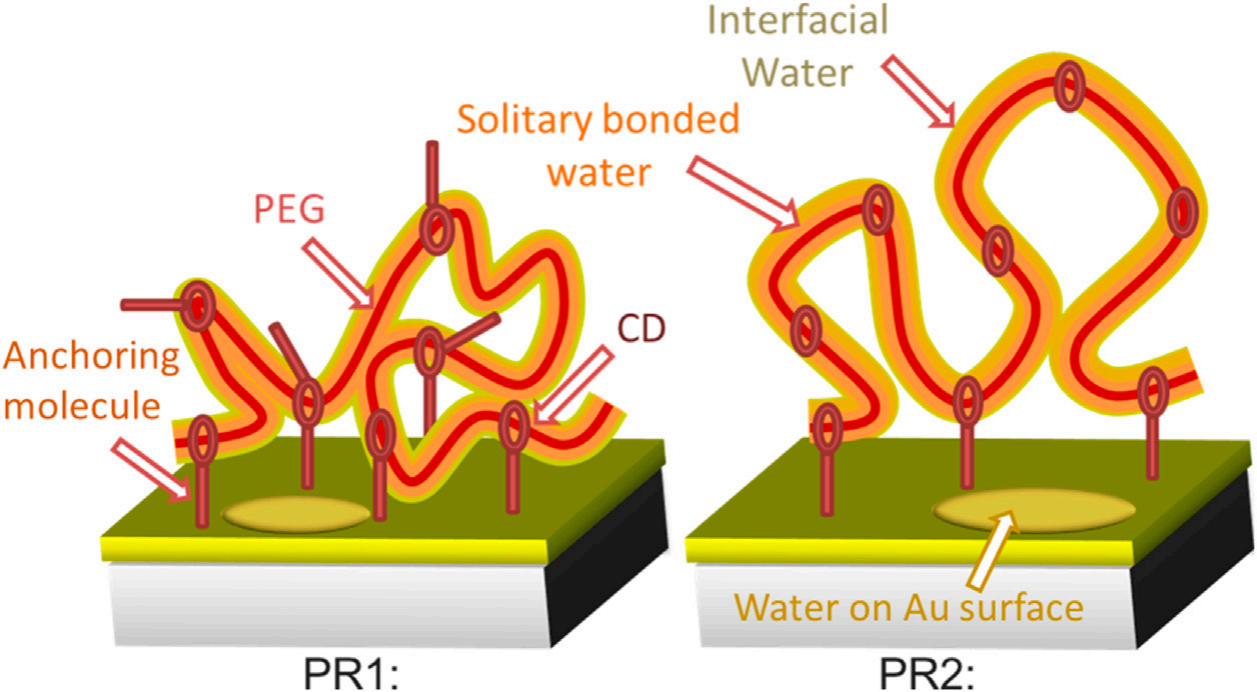
Schematic images of the surface morphology of polyrotaxane and incorporated water at RH 50%. A thin single bonded water layer (orange) surrounds the hydrophilic PEG (red string) surface. Interfacial water is present in the outer region of the incorporated water (yellow). Water molecules also present on the exposed Au surface.

In this study, due to its superb biocompatible properties and simple molecular structure, we investigated a looped poly (ethylene glycol) (PEG) to identify the hydrogen-bonded structure of water responsible for the biocompatibility (Bag and Valenzuela, 2017). Among various polymer materials, PEG is one of the most widely used polymers for biocompatible applications (Lowe et al., 2015; Bag and Valenzuela, 2017). To investigate the effect of looped morphology, we controlled the loop size by introducing multiple fixing points in a PEG chain using a polyrotaxane consisting of α-cyclodextrin (CD) and PEG chains (Scheme 1). The PEG chain can be fixed by the reaction between the dithiol end of an anchoring molecule connected to the topological ring of a CD molecule and Au substrate (Liu et al., 1999). By changing the density of the anchoring molecules and the number of fixing points in the main chain, we expected to control the size of confined spaces in the polymer loops and the amount of confined water in it. Indeed, a larger density of the fixing points reduced the volume expansion by water uptake (Kato et al., 2015).

The hydrogen-bond network of water surrounded by the looped PEG chains was investigated by monitoring the electronic structure of water using oxygen K-edge X-ray emission spectroscopy (XES), which is very sensitive to the hydrogen-bonded network of water (Tokushima et al., 2008; Yamazoe et al., 2017). By gradually increasing the humidity and monitoring O 1s XES spectra for looped PEG chains having different anchoring densities, we have successfully elucidated the structure of water inside the looped PEG and proposed the actual role of the confined water on biocompatibility.

## Materials and Methods

### Synthesis and Preparation of Anchored Polyrotaxane Samples

A hydroxypropyl-modified polyrotaxane consisting of CD and PEG (*M*_w_ = 350,000) end-capped with adamantane was purchased from Advanced Softmaterials Inc (Tokyo, Japan). Each polyrotaxane molecule had a structure with 100 CD molecules. All other chemicals and solvents were purchased from Sigma-Aldrich (Tokyo, Japan) or Wako Pure Chemical Industries, Ltd (Osaka, Japan) and used without further purification. Here is an example of preparation of lipoic-acid-introduced polyroaxane (LAPR). 325 mg of dried polyrotaxane was dissolved in 5 ml of dried *N*, *N*-dimethylformamide (DMF). 89 mg of (±)-α lipoic acid, 124 mg of 1-ethyl-3-(3-dimethylaminopropyl) carbodiimide hydrochloride, and 75 mg of *N*-hydroxysuccinimide were added in the solution. The solution was stirred at room temperature under an Ar atmosphere for 12 h. After the reaction, the solution was poured into an excess amount of acetone to reprecipitate LAPR. After drying in vacuum at room temperature, 130 mg of LAPR was obtained. We prepared two kinds of LAPRs with different density of anchoring molecules of sulfo groups per CD: high density (LAPR1) and low density (LAPR2). Each polyrotaxane was dissolved in dehydrated dimethyl sulfoxide (0.2 mg/L). Au-coated membranes were immersed in the solutions and mixed together to initiate a reaction of Au and dithiol. After overnight mixing at room temperature under an Ar atmosphere, the LAPR was anchored to an Au-coated membrane surface. Measured samples with different anchoring densities (PR1 and PR2) were obtained from solution of LAPR1 and LAPR2, respectively.

### XES Measurement of Humidity Controlled Looped PEG

**FIGURE 1:**
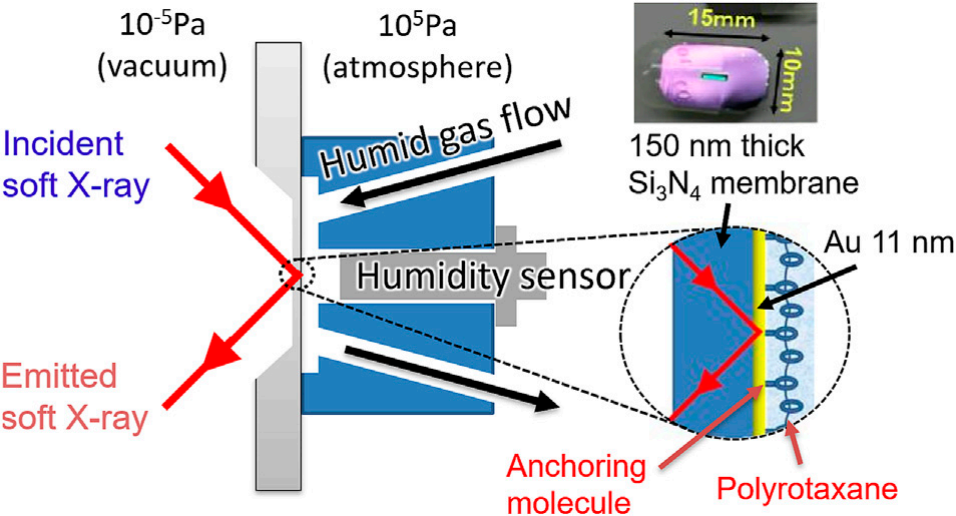
Schematic image of a sample cell for soft X-ray absorption and emission spectroscopy. Polyrotaxane polymers were fixed onto an Au/Ti-coated 150 nm thick Si_3_N_4_ membrane using an anchoring molecule and exposed to N_2_ gas flow with precisely controlled humidity.

We used the HORNET XES station (Harada et al., 2012) installed at the University of Tokyo Synchrotron Radiation Outstation beamline BL07LSU (Yamamoto et al., 2014) in SPring-8. In this system, an originally developed liquid cell separates a sample in ambient condition and the high vacuum using a 150-nm-thick Si_3_N_4_ membrane coated with 3-nm Ti and 11-nm Au layers (NTT-AT, Figure 1).

In this cell, the flow path is designed so that the humidified gas flows along the membrane, in front of which a humidity sensor was attached. By flowing a gas with precisely controlled humidity in step-wise increments, we can detect water being gradually adsorbed on the sample surface without accumulating huge background of bulk liquid water on the flow path of the humidified gas. Thus the hydrated water on the polymer can be detected without being buried in the signal from the bulk water. In the XES experiment, the analytical depth is c.a. 350 nm estimated by using an online software package at CXRO (Henke). Since the adsorbed water is only distributed in the polymer region, which is approx. 35 nm as discussed in Results and Discussion and much shallower than the detection depth, the signal detection efficiency does not vary significantly between those closer to the substrate and those in the deeper region. Roughly estimated by using the same software, the detection efficiency at the 35 nm region is about 90% of the substrate region, thus the signal near the substrate is not enhanced compared to the deeper region.

During the experiment, the temperature inside the cell was kept at 30°C, and the gas flow tube was heated at 40°C to avoid dew condensation of the humidified gas in the tube. The relative humidity (RH) of the humidified gas at the sample was precisely controlled within ±1.5% measurement error by a steam generator (HUM-1, Rigaku Corp.) that mixed vapor with dry nitrogen. Ultrapure water (Direct-Q, Millipore Inc.) was used as the steam source. When we took measurements under dry condition, dry nitrogen directly flowed into the cell to keep the relative humidity below 10%. This dry gas effectively prevents water adsorption on the sample and enables us to obtain only signals from oxygens in the polyrotaxane polymer film and in the Si_3_N_4_ membrane.

All O 1s XES measurements were performed at an ionization condition with an incident energy of 550 eV and total energy resolution of 0.15 eV. At each humidity condition, three or four XES spectra were obtained and summed after a consistency check, where the exposure time for each spectrum was 30 min, while for those with higher H_2_O signal intensity from pure water and at RH 95% in PR1 and PR2, only one 30 min spectrum was obtained. The obtained XES spectrum at dry condition (<RH 10%) was subtracted from the spectrum at each humidity condition. Raw XES spectra are shown in Supplementary Figure S1. To estimate background contributions from water adsorbed on the substrate 11-nm Au layers, an XES spectrum of water adsorbed on the same Si_3_N_4_ membrane without PR1 and PR2 samples was also obtained.

## Results and Discussion

**FIGURE 2:**
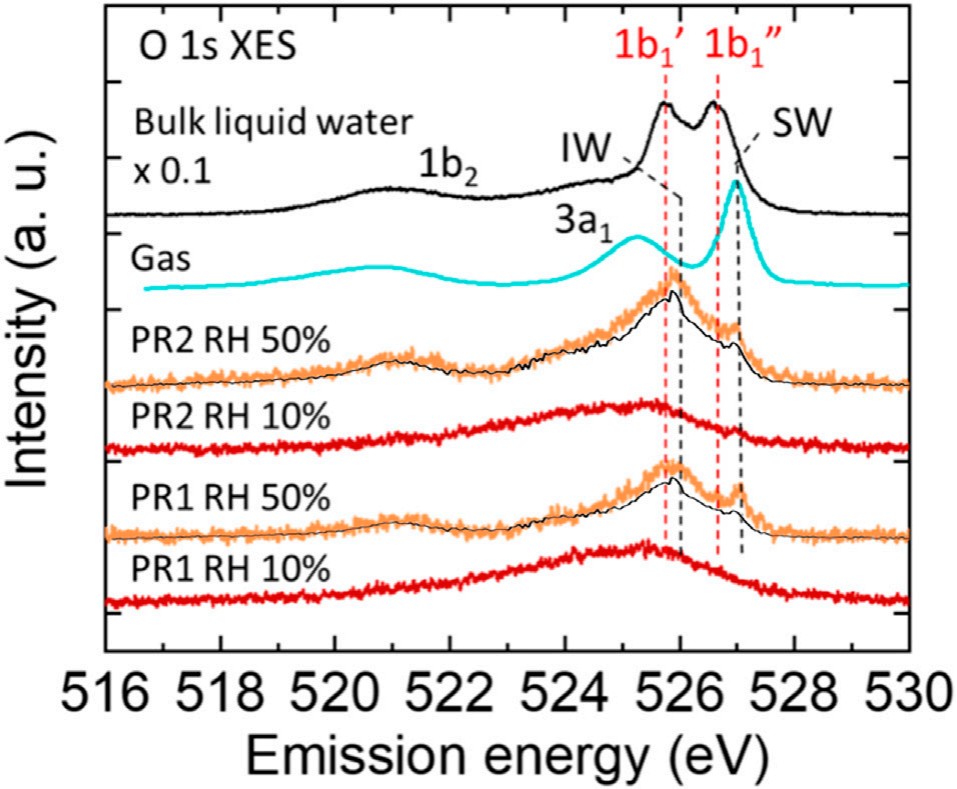
O 1s XES spectra of water in the polyrotaxane polymer film at RH 50% (orange) obtained by subtracting the corresponding polyrotaxane spectra at a dry (<RH 10%) condition (red). The intensity of the bulk liquid water spectrum (black) is multiplied by 0.1. The gas phase spectra (blue) is obtained from other report (Weinhardt et al., 2012). The background XES contribution from water adsorbed on the Au surface is also plotted (black line) on each RH 50% spectrum.

Figure 2 shows the XES spectra of water in the polyrotaxane polymer film at RH 50% (orange plots). They are subtracted by the corresponding RH 10% (dry condition: red plots) spectra showing oxygen signals from the polyrotaxane and Si_3_N_4_ membrane. For comparison, the XES spectrum of bulk liquid water multiplied by 0.1 (black line) and vapor gas (blue line) (Weinhardt et al., 2012) are also added. The bulk water spectrum is an integration of signals from a probing depth of 300 nm, and is multiplied by 0.1 because it is much more intense than adsorbed water, from a few tens of nm on the substrate surface. The bulk liquid water spectrum showed two sharp 1b_1_-derived peaks, which correspond to tetrahedrally coordinated (1b_1_′) and distorted (1b_1_″) hydrogen-bonds (Tokushima et al., 2008). The XES spectra of water in the polyrotaxane polymer films have a very different profile around the 1b_1_ peaks from the bulk liquid water spectrum, indicating that incorporated water molecules in the films have hydrogen-bonded configuration totally different from that of bulk liquid water.

The PR samples all have a 527.0 eV peak, which is higher than the 1b_1_″ peak of bulk liquid water (526.6 eV), and very close to the 1b_1_ peak of water vapor reported as 527.1 eV (Weinhardt et al., 2012). However, the peak around 527.0 eV often appears in monolayer water molecules directly bonded to metal surfaces, where the water molecules are aligned with direct O bonding or H-down bonding to the metal surface (Schiros et al., 2010). At present, there are two possibilities for the 527.0 eV peak. One is water molecules directly bonded to the PEG chain rotating to point their H atoms to the O atoms in the PEG units (Zhao et al., 2005). The other is water adsorbed on the exposed Au surface not covered by PR1 and PR2 molecules. For the latter contribution the XES spectrum for water adsorbed on the Au surface is also plotted in Figure 2 as black solid line on the RH 50% spectra for PR1 and PR2. The intensity is normalized not to exceed the PR1 and PR2 spectra at all energies. Approximately 80% of water adsorbed at RH 50% may be ascribed to water on the Au surface, which corresponds to the relative amount of 0.667 and 1.256 for PR1 and PR2, respectively (see Supplementary Table S1 in the Supporting Information). The remaining water molecules should be adsorbed on ether O atoms in the PEG units, which was reported to be monomeric (singly bonded) at RH 50% by using infrared spectroscopy (Kitano et al., 2001). Accordingly, we denote the peak at 527 eV a single bonded water (SW) peak, as shown in Figure 2.

The PR samples have a primal peak at 525.9 eV which is higher energy than the 1b_1_′ peak of bulk liquid water (525.7 eV) and the 1b_1_ peak of the ice phase reported as 525.6 eV (Gilberg et al., 1982). Similar peak close to the 1b_1_′ peak has been observed in a humidified polyelectrolyte brush (Yamazoe et al., 2017), where absorbed water is surrounded by crowded polyelectrolytes and had a distorted tetrahedrally coordinated (ice-like) hydrogen-bond structure even at room temperature. In previous reports ice-like structures were also observed on PEG self-assembled monolayer (SAM) (Anderson and Ashurst, 2009) and UV/ozone-treated SiO (Asay and Kim, 2005) by using attenuated total reflection infrared spectroscopy (ATR-IR), where water molecules were aligned on the functional groups and formed a few water layers with a tetrahedral network. Kitano et al. (2001) have analyzed ATR-IR results with an ab initio molecular orbital (MO) calculation method and hybrid density functional method. They reported a part of water adsorbed on the ether oxygen forms a dimer structure with other water molecule or bridges other PEG chain to form a hydrogen bonded network of water molecules at 50% RH. Daley and Kubarych found by calculation that water molecules form hexagonally arranged water structures connecting to adjacent PEG oxygen atoms (Daley and Kubarych, 2017). This hexagonal motif is the primitive building block of an ice I_h_ structure, although water molecules surrounding the PEG chain clearly lack the long-range ordering due to the twisted conformation of the underlying polymer. They also mentioned the tight integration of the PEG/water interface results in a highly “structured” hydration layer. Our obtained peak at 525.9 eV may indicate that the adsorbed water molecules form highly structured hydrogen bonds with other water molecules and/or polymer chains to form a tetrahedrally coordinated network. We call such tetrahedrally coordinated water as “interfacial water” (IW) and denoted the 1b_1_ peak at 525.9 eV as the IW peak.

PR1 and PR2 showed different IW peak intensities at RH 50%. Polyrotaxane molecules with a larger number of anchoring molecules (= PR1) should have a less volume and decreased water content [17]. Accordingly, PR1 shows weaker intensity of the IW peak than that of PR2, as shown in Scheme 1.

**FIGURE 3:**
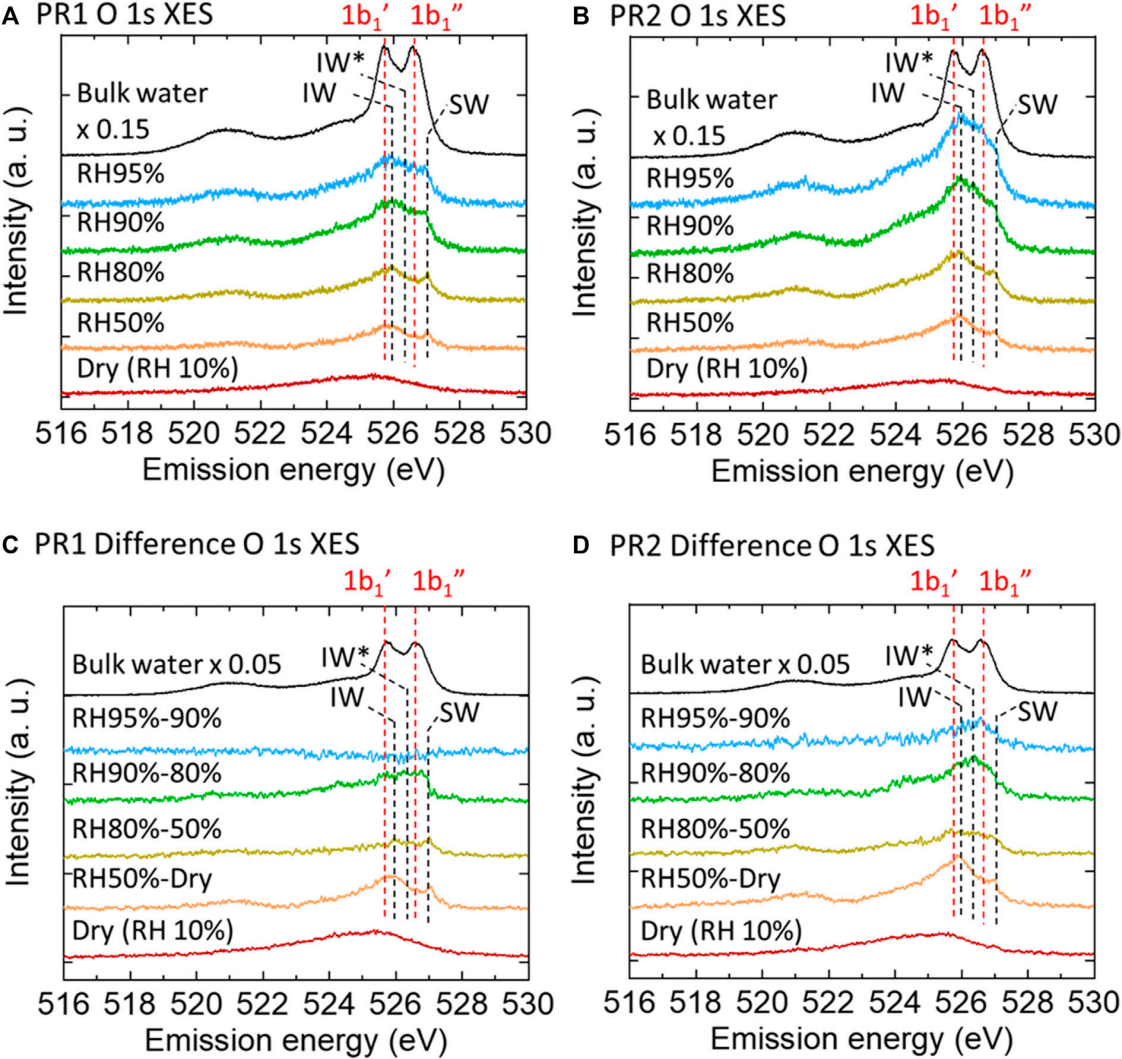
O 1s XES spectra of PR1 **(A)** and PR2 **(B)** polyrotaxane polymer films at various humidities obtained by subtracting the dry polymer spectra. Dry (RH 10%) and pure water spectra are the raw data. Each difference spectrum **(C,D)** was obtained by subtracting the neighboring lower-humidity spectrum.

Figures 3A,B show O 1s XES spectra of PR1 and PR2 under relative humidities of 50, 80, 90, and 95%. They are all subtracted by the corresponding RH 10% (dry condition) spectra. To extract the character of deposited water at each humidity step, difference spectra between the XES spectra of neighboring humidity conditions for PR1 and PR2 are shown in Figures 3C,D.

Assuming that the contribution from water on the Au surface may be proportional to the humidity, the amount of water adsorbed on the PR1 and PR2 samples can be estimated by subtracting those contributions from water on the Au surface (shown in the bottom two columns in Supplementary Table S1). It is expected that almost all the oxygen moieties of PR1 and PR2 are covered by water molecules above RH 90% as indicated by the value greater than unity. Since the anchoring molecules are hydrophobic, the adsorbed water covering the polyrotaxane chain mainly exists on the hydrophilic PEG and CD surface under humid conditions. Here, the CD ring can slide on the PEG chain, but we do not take care of the sliding effect on the adsorbed water molecules on the PEG chains since those adsorbed water molecules are dynamically adsorbed and desorbed typically in the time scale of 10 ps (Ensing et al., 2019), while the time scale of the sliding of α-CDs along the PEG chains is on the order of 0.1^−1^ ms (Zhao et al., 2005). Therefore, we consider that the motion of α-CDs does not affect the adsorption dynamics of water on the PEG chains. At higher relative humidity than 90%, additional water layer grows. PR2 polymer absorbs more water than PR1 in the swollen structure as shown in bold fonts in Supplementary Table S1. Since the density of PEG chains should be almost identical between PR1 and PR2, the increase in the water uptake in PR2 at higher humidity is surely coming from water molecules confined in the polymer loop as discussed in the following.

As already discussed above, for both PR1 and PR2, the RH 50% spectra showed an increase of the IW and SW peaks after humidification. From RH 50–80%, the XES spectrum showed only a slight increase. Upon the humidification process on a hydrophilic Si-OH surface, Asay and Kim (2005) reported that water molecules form bulk-liquid like water layers on the tetrahedrally coordinated hydrogen-bonded layers. In the present case, the spectral difference between RH 50 and 80% would show a small increase of water having a variety of hydrogen-bonded structures on the tetrahedral interfacial water layer. At RH 80% at room temperature, the vapor pressure of water is 3.1 kPa, which is comparable to the osmotic pressure of PEG solution with 3,000 molecular weights when the PEG/water ratio is 1:2 (Money, 1989). The average chain length between adjacent CDs in our polyrotaxane is 3,000 molecular weights, which is roughly consistent with our XES results in terms of the correlation between the molecular weight and the water content. PEG is well known as a crystalline polymer and generally has a helical conformation in the film state (Gemmei-Ide et al., 2006; Oparaji et al., 2016). The crystallinity of the PEG film was evaluated to be around 70% irrespective of its molecular weight (Kitano et al., 2001), and the other region has amorphous form. Water molecules in the crystalline phase of the PEG film might be localized in the amorphous region of the PEG chain because gaseous water diffuses mostly to the amorphous region in solid PEG polymers (Gemmei-Ide et al., 2006). Oparaji et al. (2016) reported that crystallinity of PEG was almost constant up to around RH80%, and this high ratio of crystallinity could explain the limited water absorption.

**SCHEME 2:**
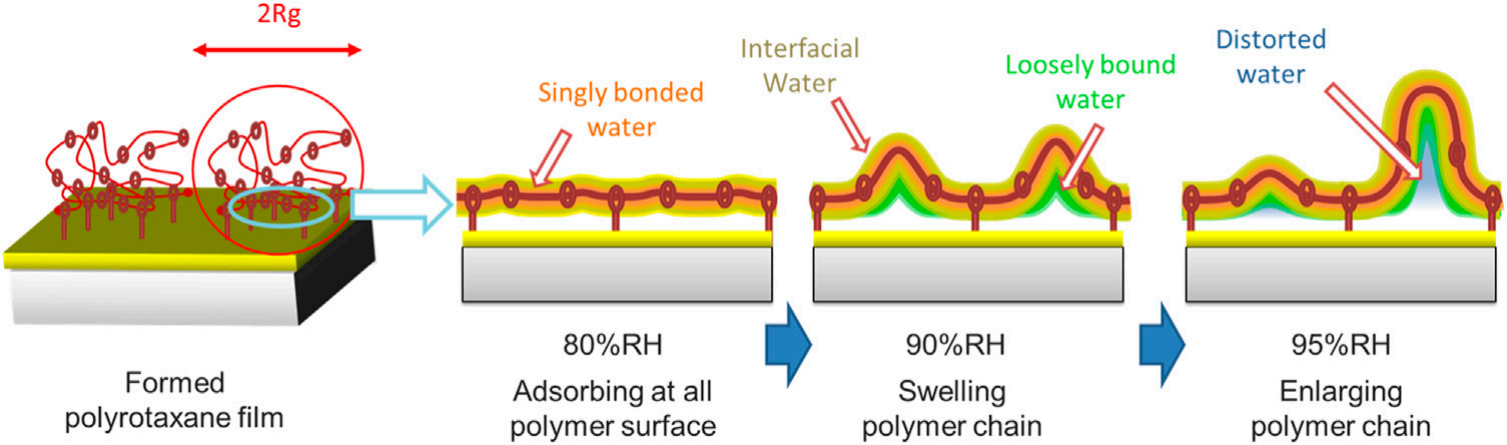
Schematic images of the morphology changes of polyrotaxane and water. A thin directly bonded water layer (light blue) and interfacial water surround the hydrophilic PEG surface. Loosely bound water (green) and distorted water (orange) are located inside the large interfacial water region.

In contrast, at RH 90%, the intensity of the difference spectrum in the 1b_1_ region rapidly increases again. The degree of PEG crystallinity decreases with increasing humidity, especially from around 80%RH. Operaji et al. (2016) have demonstrated that the polyethylene oxide (PEO)-water interactions become more favorable with increasing humidity. Even though the PEO was of high molecular weight with highly entangled chains, it became a soft gel with no mechanical strength at high relative humidity. Our sharp upturn in sorption above RH 80% suggests that PEO/water interactions overcome crystallite restraints and lead to dissolution. These spectra show a broad peak centered at around 526.3 eV, which is higher energy than the IW peak at 525.9 eV. This is explained by an increase in the number of dangling bond defects in the hydrogen-bonded network (Zhovtobriukh et al., 2018). Such dangling bond defects are observed in a PEG solution; water molecules surrounded by the PEG pseudo-network have distorted hydrogen-bonded structure, which has 1.0 less hydrogen-bonds per monomer unit of a polymer (Kitano et al., 2000). Accordingly, we consider that the absorbed water during the increase from RH 80% to RH 90% is a loosely bound water which has a hydrogen-bonded structure distorted by the nearby polymer chains as in the PEG solution. We call this 1b_1_ peak at 526.3 eV the IW* peak. The corresponding 1b_1_ peaks are tabulated in Table 1. The intensity of the difference spectrum in PR2 is larger than that in PR1. When polyrotaxane absorbs a large amount of water, the polymer chain swells to a large volume (Bin Imran et al., 2014) and the size of the PEG loop develops due to the sliding CD rings. Therefore, above RH 90%, slack in the polymer chain would be enlarged and further increase the amount of absorbed water (Scheme 2).

**TABLE 1:** Detailed peak assignments of the 1b_1_ peak corresponding to each combination of energy and water structure.

Peak	1b_1_′	IW	IW*	1b_1_′′	SW
Emission energy (eV)	525.7	525.9	526.3	526.6	527.0
Water structure	Bulk-liquid Tetrahedrally coordinated	Interfacial	Loosely bound	Bulk-liquid Distorted	Single bonded

At RH 95%, the PR1 1b_1_ peak does not increase because of the limited space for water absorption, while the PR2 1b_1_ peak continued to increase, suggesting that additional water molecules can be stored in the large slack of the polymer loop. The PR2 1b_1_ peak has a peak at 526.6 eV, which is commonly observed in bulk-liquid water as a 1b_1_″ peak. Many researchers have argued that bulk-liquid water contains two types of hydrogen-bonded structure: tetrahedrally coordinated and distorted. The former would correspond to the 1b1′ peak and the latter to the 1b1″ peak (Gallo et al., 2016). The 526.6 eV peak in PR2 suggests that the absorbed water has distorted hydrogen-bonded structure close to the 1b_1_″ component in bulk-liquid water. However, the 1b1′ was not increased. The 1b_1_′ peak originates from water in tetrahedral-like patches with a dimension of 1–1.4 nm in dynamic equilibrium (Huang et al., 2009). The absence of the 1b_1_′ peak is possibly due to the limited space in polyrotaxane chains. In fact, the radius of gyration (Rg) of the PEG samples are estimated to be ∼35 nm from the equation Rg (in nm unit) = 0.0215 *M*_w_^0.58^ (Devanand and Selser, 1991) in a solution, where *M*_w_ is the molecular weight of PEG. Because the PEG chain is flexible and bendable and some water molecules are bridging neighboring polymer chains, the spaces in some polymer loops might be decreased to less than 2 nm. Therefore, our presumption that water is confined within a space of a few nanometers seems reasonable. This is analogous to the case for mesoporous silica where differential scanning calorimetry measurements showed no freezing and melting point when the pore size of the mesoporous silica was less than 2.1 nm (Kittaka et al., 2006). This is explained that the pore diameter is less than the critical point of the nucleation diameter of 2.2 nm. We expect that the hydrogen-bonded structure of water will be significantly modulated when confined in a polymer loop.

Using various techniques the structure of hydrating water around biocompatible polymers has long been studied. Morita et al. (2007) conducted time-resolved ATR-IR on PMEA, and found that the freezing-bound water, which has hydrogen-bonding to the polymer with an intermediate strength between ice and bulk-liquid water, is likely to function as a biocompatible interface. If their discussion can be applied also to PEG, interfacial water or loosely bound water might be the origin of biocompatibility. Kittaka et al. (2006) found that water confined in a small space shows a cold crystallization property, which is also observed in biocompatible polymers (Tanaka and Mochizuki, 2010). Ide et al. (2003) estimated an extinction coefficient of the OH stretching band of absorbed water on a PMEA biocompatible polymer with ATR-IR measurement, and concluded that a cold crystallization of water is generated by caging water molecules in small spaces formed by the polymer chains. These previous studies indicated that water molecules confined in a narrow space surrounded by polymers could have a relation to the biocompatibility. Indeed, Yamada et al. (2012) reported that a polyrotaxane with looped PEG chains exhibited better biocompatibility than that with a linear one. We expect that the amount of water incorporated into the looped PEG, which may contribute to biocompatibility, could be controlled by changing the loop size according to the anchoring density.

## Conclusion

Specific hydrogen-bonded structure of incorporated water into polyrotaxane polymer films was investigated by O 1s X-ray emission spectroscopy. By changing the density of anchoring molecules that determine the number of fixing points in the main chain of the polyrotaxane polymer, we controlled the size of the confined space in the large slack of the polymer loop and the amount of absorbed water in it. The XES spectra showed that at low humidity, water molecules directly bonded to the polymer formed a tetrahedrally coordinated hydrogen-bond, while increasing the humidity, loosely bound and distorted hydrogen-bonded water dominated. In particular, at high humidity above RH 90%, uptake of water having distorted hydrogen-bonded structure with the peak around 1b_1_″ was observed. Intriguingly, the XES spectrum of the incorporated water completely lacks the tetrahedral 1b_1_′ peak, suggesting that the incorporated water is confined in the polymer loop where tetrahedral-like patches could not exist. Because confined water in a similar space in biocompatible polymers shows cold crystallization, the incorporated water in the polymer loop seems to be one of the origins of the superior biocompatibility of the looped polyrotaxane. Our study demonstrated that the amount of the confined water which would contribute to biocompatibility can be controlled by changing the anchoring density of polyrotaxane.

## Data Availability

The original contributions presented in the study are included in the article/Supplementary Material, further inquiries can be directed to the corresponding authors.
