# Supplementary material for: Hydrogen-Bonded Structure of Water in the Loop of Anchored Polyrotaxane Chain Controlled by Anchoring Density
Source: Front Chem. 2021 Oct 18;9:743255. doi: 10.3389/fchem.2021.743255 (PMC8577270; doi:10.3389/fchem.2021.743255)
Supplement: Supplementary file 1 [file DataSheet1.docx]

Supplementary Materials


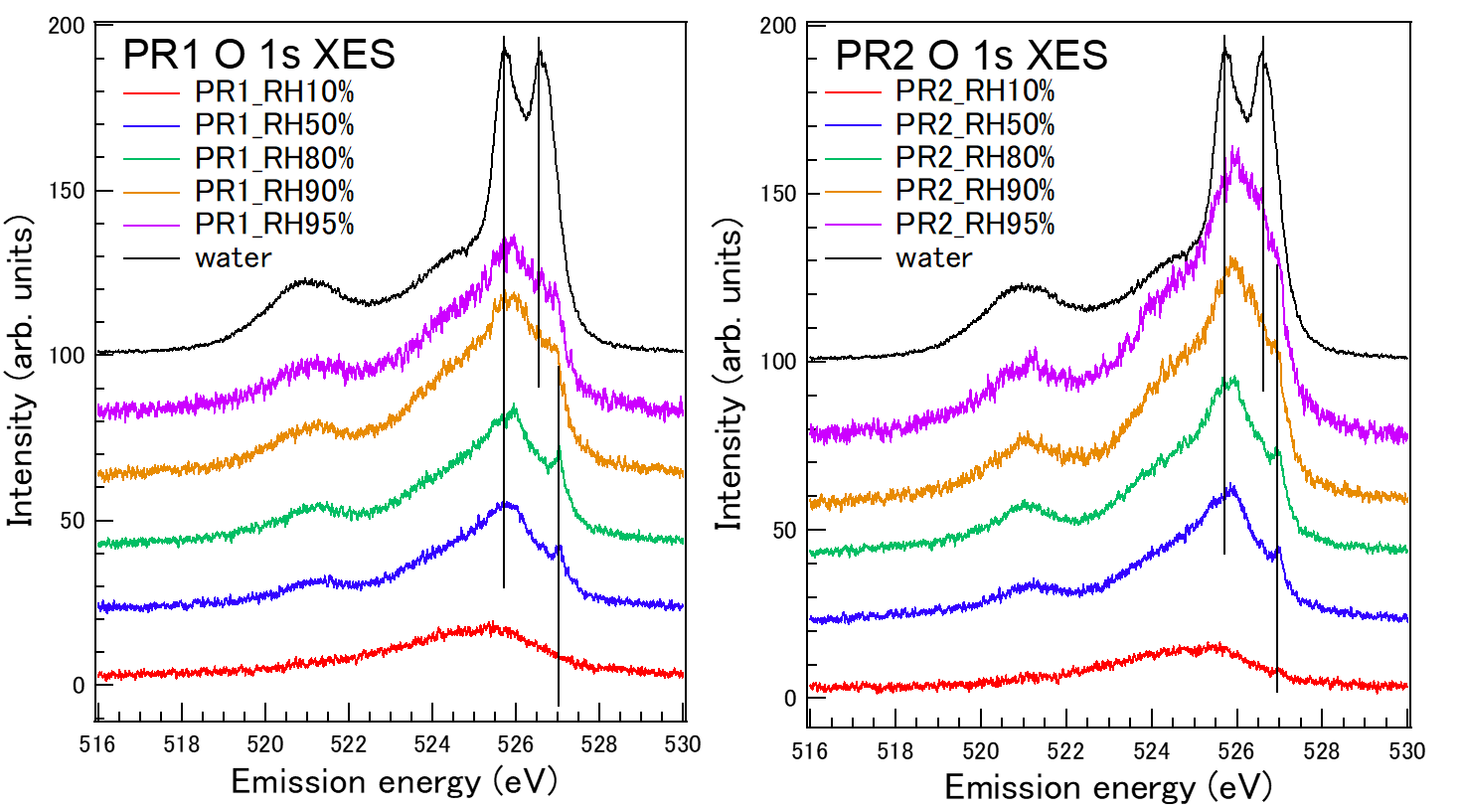


Figure S1. Raw data of PR1 and PR2 XES spectra.

Table S1. Relative amount of the total adsorbed water, water adsorbed on Au and their subtraction at each humidity relative to the dry condition.

| Relative humidity | RH 50% | RH 80% | RH 90% | RH 95% | Pure water |
| --- | --- | --- | --- | --- | --- |
| PR1 relative amount to dry | 0.836 | 1.374 | 2.262 | 2.083 | 4.234 |
| PR2 relative amount to dry | 1.570 | 2.365 | 3.769 | 4.507 | 4.880 |
| Water adsorbed on Au for PR1 | 0.667 | 1.067 | 1.201 | 1.267 |  |
| Water adsorbed on Au for PR2 | 1.256 | 2.010 | 2.261 | 2.386 |  |
| PR1 – [water adsorbed on Au] | 0.169 | 0.307 | 1.061 | **0.816** |  |
| PR2 – [water adsorbed on Au] | 0.314 | 0.355 | 1.508 | **2.121** |  |
